# Small-scale forestry and carbon offset markets: An empirical study of Vermont Current Use forest landowner willingness to accept carbon credit programs

**DOI:** 10.1371/journal.pone.0201967

**Published:** 2018-08-14

**Authors:** Alisa E. White, David A. Lutz, Richard B. Howarth, José R. Soto

**Affiliations:** 1 Environmental Studies Program, Dartmouth College, Hanover, New Hampshire, United States of America; 2 School of Natural Resources & The Environment, The University of Arizona, Tucson, Arizona, United States of America; University of Waterloo, CANADA

## Abstract

This study investigates the preferences of small forest landowners regarding forest carbon credit programs while documenting characteristics of potentially successful frameworks. We designed hypothetical carbon credit programs with aggregated carbon offset projects and requirements of existing voluntary and compliance protocols in mind. We administered a mail survey to 992 forest landowners in Vermont’s Current Use Program utilizing best-worst choice, a novel preference elicitation technique, to elicit their preferences about these programs. We found that small forest landowners see revenue as the most important factor in a carbon credit program and the duration of the program as the least important factor. Landowners reported that shorter program duration, higher revenue, and lower withdrawal penalties positively impact their willingness to accept forest carbon credit programs. Notably, our study includes carbon credit program implementer as a key program attribute, allowing us to quantify landowners’ tradeoffs between non-profit, for-profit, and government organizations. Overall, we found that landowners significantly prefer working with a non-profit organization. Based on monetary estimates of willingness-to-accept compensation, our results suggest that aggregated forest carbon offset projects incorporating small forest landowners could be piloted successfully in Vermont by non-profit organizations while maintaining relatively strict guidelines of existing carbon offset protocols.

## Introduction

Forest ecosystems have significant potential to mitigate greenhouse gas emissions in the atmosphere by acting as reservoirs that accumulate and store carbon[1,2,3]. These reservoirs of carbon storage can be quantified and certified as carbon offsets under the requirements of forest carbon offset protocols and then be integrated within voluntary or compliance carbon offset markets. In the case of compliance markets, such offsets are generally purchased by emitters that must comply with mandatory, legally binding emissions reductions that are rigorously supported by legislation and legal frameworks[3]. In the United States, the California Air Resource Board (CARB) manages the only compliance protocol that has granted emissions credits for forest carbon offsets [4]. Under CARB’s protocol, forest carbon offsets can be generated from forests across the country that store carbon above a regional baseline, with the potential for additional offset generation with documented net carbon storage over time [5]. Over 200 forest offset projects have been proposed or implemented in CARB’s program since 2014 [6].

Generally, forest carbon offset projects go through three distinct processes in order to create offsets: a feasibility/baseline analysis, a verification/certification process, and routine monitoring of forest carbon stocks. Both the feasibility study and the verification/certification process are costly and risky as there is no guarantee that the revenue generated from auctioning the carbon offsets will be enough to outweigh the cost of project implementation [4,7,8]. Once offsets are sold, monitoring and enforcement costs over the 100-year life of a CARB forest carbon offset project can be between $250,000 and $500,000[6–8]. As a result, if the project does not uphold its requirements, whether intentionally or unintentionally, the offsets can be invalidated in a “reversal” and a withdrawal penalty may be levied. Given this risk and uncertainty, small forest landowners with parcels of forest land of less than 4000 acres on which they can generate offsets are less inclined to enter the program. Consequently, large forest land holdings of 4000 acres or more make up the majority of forest carbon offset projects in California’s market[6].

### Program feasibility and implementation

Several feasibility studies have been conducted that examine small-scale forest owner participation in forest carbon offset markets in the United States[9–15]. Prior studies have found that a lack of financial motivation, upfront management plan, and certification costs often deter small forest landowners from engaging with the carbon market[16,17]. In addition, several researchers have pointed out that limited revenue from the carbon offset market, early withdrawal penalties, and long contract lengths deter small forest landowners from participating[9–11]. For instance, Dickinson *et al*. found that up to 43% of landowners would sign up for a carbon credit program with a five-year commitment, no management plan, $30 per acre in annual revenue, and no withdrawal penalty[9]. Yet, this program may be unrealistic given that CARB’s forest carbon offset protocol requires landowners to commit for 100 years to the program and have a management plan as part of generating a forest carbon offset project. Even voluntary carbon offset protocols such as the Verified Carbon Standard (VCS) have contract lengths of 20-years at the minimum[18]. Similarly, Markowski-Lindsay *et al*. found that stricter programs requiring a management plan, 30-year commitment, and withdrawal penalty, paying $10 per acre per year would only see a 4% enrollment[10]. In addition to programmatic factors, small property sizes prevent small forest landowners from participating in California’s emerging carbon market, particularly in the Northeast, where family forest owners live on an average of just 50.66 acres of forest[8]. With approximately 61% of forest land in Vermont in the hands of family forest owners, low participation removes large quantities of valuable forest carbon from offset markets[19]. Therefore, it is important to better understand small landowners’ preferences for forest carbon credit protocol design in order to encourage their participation in the carbon offset market.

Prior feasibility studies have primarily used logit and probit models to analyze the impact of carbon credit program characteristics on landowner enrollment in the program. For instance, Fletcher *et al*. explored the willingness of Massachusetts private forest landowners to engage with carbon credit programs by asking landowners to rank hypothetical carbon credit programs with varying management plan requirements, time commitments, expected payment, and withdrawal penalty [11]. These results suggest that ratings of the hypothetical program increase with both payment and commitment length, but decrease with the inclusion of a withdrawal penalty. Following [11], Markowski-Lindsay *et al*. utilized a probit model to analyze the impact of additionality requirements and the percent of land that the owner must enroll in the program, among other factors, on the stated participation rate in the program [10]. In this instance, the authors found that landowners preferred a program with higher revenue, no withdrawal penalty, and shorter contract lengths. Miller as well as Simpson and Li constructed surveys in Texas and the Lake States and presented respondents with hypothetical forest carbon credit programs with varying revenue levels and contract lengths[12,13]. The latter found that an $18 per acre per year compensation was required to ensure 50 percent participation. More recently, Soto *et al*. (2016) explored new aspects of small forest landowner willingness to participate in forest carbon credit programs through the application of best-worst choice (BWC) to evaluate forest owners willingness to participate[14]. Soto *et al*. found that revenue was the most important attribute to small forest landowners while contract length of the program was the least important attribute to forest landowners when evaluating forest carbon credit programs[14]. While many other studies focus on the impact of program characteristics on enrollment in the program, our study provides unique insight as to landowner willingness to accept specific attributes of carbon credit programs.

Although some prior studies on carbon credit programs for small forest landowners acknowledge the potential importance of a program implementer[10,14], these studies do not focus on landowner preferences for program governance. Our study seeks to address this gap in the literature by including the program implementer in the choice scenarios for this study. However, in the area of forest certification and management, a variety of studies have examined how forest landowners interact with government and non-profit organizations to guide management decisions for their forest land. As a whole, landowners tend to distrust direct government involvement on their land during forest certification and dislike the bureaucratic structures associated with government involvement[20,21]. Similarly, state government cost share and tax incentive programs show mixed success—Indiana’s cost share program has low participation among landowners and, across the Northern U.S., state tax incentive programs for forest management are well known to landowners but have limited appeal due to direct government involvement and withdrawal penalties, among other factors[22,23]. Vermont’s Use Value Appraisal Program is one such government tax incentive program that offers a tax break for landowners but struggles to maintain membership in part due to development pressure and forest parcel fragmentation[24]. As a whole, the government programs with clearer goals for forest management tend to have the largest tax and administrative benefits but also more strict requirements for management plan and withdrawal penalty[24].

On the other hand, forest certification programs run by non-profits including Forest Stewardship Council (FSC), Sustainable Forestry Initiative (SFI), and the American Tree Farm System (ATFS) are seen as a viable strategy for landowners seeking to sustainably manage their forest[25]. ATFS focuses primarily on small forest landowner certification and requires that landowners have a management plan that adheres to ATFS environmental standards, pass an inspection by an ATFS forester, and agree to third party audits[26]. FSC, while originally focused on large landowners, has allowed small forest landowners to become certified by agreeing to be a part of a larger forest management plan or as part of a group certification[21]. This process of group certification or joining a larger certification plan is similar to an aggregated carbon offset program insofar as landowners agree to manage their forest in accordance with requirements dictated by FSC as part of a management plan involving multiple landowners. Although FSC or ATFS certification requires restrictions on landowners’ forest management practices, landowners perceive both economic and intrinsic value to these certification programs. Certification programs can give landowners a sense of well-being and intrinsic benefit from protecting the forest land as well as give landowners who sell timber and other forest products a price premium[21]. Beyond forest certification, landowners have become increasingly willing to collaborate with non-profit land trusts on conservation easements[27,28]. Private companies have had limited involvement in the forest certification sector, but approximately 26% of small forest landowners have harvested timber and engage with companies and other landowners in order to sell this timber[29].

### Project aggregation

While many feasibility studies ask individual landowners about their preferences for forest carbon credit programs, an aggregation of smaller parcels of land into projects of a larger size will likely be necessary to allow any sort of widespread participation of small forest landowners in the carbon offset market[8]. However, aggregation has not yet been mainstreamed into CARB’s compliance offset protocol; aggregated projects are only allowed if aggregated parcels have the same baseline and inventory for the project, have joint verification, and do not cross more than two ecosections, geographic units defined by the U.S. Forest Service that are based on native tree species.[14]. In this study, we elicit respondent preferences for participation in forest carbon credit programs designed with aggregation in mind. Since project aggregation is not yet developed in CARB’s protocol, we drew on project aggregation requirements from the Climate Action Reserve (CAR) and American Carbon Registry (ACR) while designing the hypothetical carbon credit programs in this study. Aggregated projects, like projects without aggregation, must perform feasibility/baseline analysis, go through the verification/certification process, and routinely monitor forest carbon stocks. The project aggregator typically manages all aspects of this process, reducing or eliminating the need for individual landowners to coordinate directly with one another. In CAR’s protocol, the aggregator must be certified and is responsible for maintaining contracts with individual forest landowners [30]. In order to participate in an aggregated carbon credit project, the landowner must sign a contract with the aggregator to manage their forest land according to an agreed upon management strategy for a set duration of time. The contract length of projects varies from 20 to 100 years, depending on the forest carbon offset protocol in question[18].

As a whole, project aggregation helps lower the two major transaction costs to carbon offset projects for small forest landowners: establishing a baseline and verifying the project throughout its life[8,31]. First, aggregation can lower the upfront costs to each landowner in the baseline analysis process. CAR, for one, allows landowners to reach the target sampling error for baseline carbon stocks of +/-5% of the mean at the 90% confidence level across the entire project area, reducing the number of plots of forest per landowner that need to be surveyed and cutting inventory costs by up to 90% for each landowner[31]. In this process, CAR still requires each landowner to complete an inventory, but the ACR further reduces transaction costs by allowing the project aggregator to organize a shared baseline and forest inventory for the project[31]. For verification, the aggregator is responsible for choosing a single verification body for the project and coordinating mandatory site visits, pursuant to individual contracts signed between the aggregator and the landowner [30]. As a whole, CAR’s aggregated protocol requires fewer verification visits over time for each individual landowner within an aggregated project, reducing the burden on landowners[31]. Finally, monitoring of carbon stocks and forest land is typically coordinated by the aggregator as part of the repeated verification process [30].

With any carbon offset project, there is a risk of reversal and invalidation of credits. Project reversal occurs when the carbon that the project intended to store is released back to the atmosphere due to fire, storms, widespread tree death or destruction, landowners not managing their land to meet project requirements, or other disturbances[32]. Generally, the more landowners there are involved in an aggregated project, the higher the risk of an individual leaving the program, which could lead to a reversal. However, a project aggregator can help protect against landowner withdrawal from the project and the risk of reversal in a number of different ways. First, both CARB and CAR’s carbon offset protocols require a buffer pool of credits in the case of a reversal[18]. In general, the program withdrawal penalty is meant to deter voluntary reversals due to landowner withdrawal. In addition, CAR requires that landowners put their land into a conservation easement to further ensure commitment to the program for the entire duration of project[16]. ACR places control over the risk of project reversal squarely in the hands of the aggregator. If one landowner experiences a reversal on his/her land, the aggregator is allowed to find replacement credits, thereby shielding other landowners in the project from the risk of a full project reversal and invalidation of the carbon credits[31]. In general, landowner compliance with forest management practices and participation for the entire project duration is important for successful aggregated carbon offset projects. Furthermore, aggregated projects may be more viable and successful where landowners see the value in working together to conserve land and generate a carbon offset project. However, this study anticipates that the aggregator will play a pivotal role in encouraging small forest landowner participation in the carbon market by reducing the upfront costs to participating and, notably, taking on the reversal risk that could deter landowner engagement. We assume for the purpose of this study that the withdrawal penalty from the carbon credit program is the primary cost that landowners would face from voluntarily or involuntarily violating their carbon credit program contracts. The aggregator would set the withdrawal penalty for each landowner in the aggregated project based on the perceived risk of withdrawal of the landowners, among other factors in determining reversal risk.

### Goals of this study

This study focuses on potential carbon offset market participation among forest owners in Vermont’s Use Value Appraisal or “Current Use” program. Given their willingness to actively manage forest and comply with monitoring and management requirements, Vermont Current Use forest landowners may be a target population for expanding the forest carbon offset market to small forest landowners and piloting forest carbon offset project aggregation. Primarily, we seek to better understand what aspects of carbon offset projects (which we also refer to as “forest carbon credits”) are most important to these landowners. Additionally, we look to understand how these landowners view institutions that would manage aggregated forest carbon offset projects, how much compensation landowners require to participate in these projects, and, in a broader sense, if carbon credit programs for small forest landowners are viable in the state of Vermont and beyond. Unlike prior carbon credit program feasibility studies, we position the program implementer/project aggregator as an integral feature to the carbon credit program. This allows us to understand landowner program governance preferences in the specific context of the carbon offset market. We conducted a survey of 992 forest landowners utilizing best-worst choice (BWC) to address these research topics. BWC is a recent innovation in best-worst scaling (BWS), a technique first published by Louviere [33]. BWC produces estimates of both traditional discrete choice experiments (DCEs) and BWS. With the BWC technique we are able to analyze not only landowner willingness-to-accept the program as a whole, but also willingness-to-accept specific carbon credit program attributes. We also analyze if there are different preferences for these attributes among demographic groups within the Vermont Current Use population.

## Materials and methods

### Study area and survey overview

We chose the state of Vermont as our study area because of its well-established Use Value Appraisal (UVA) program, commonly referred to as “Current Use.” The Current Use program allows forest and agricultural land to be appraised for taxation based on its forestry or agricultural value rather than potential for development[34]. In order to enroll, landowners must have at least 25 acres of land and must have an active 10-year forest management plan that includes timber management on at least 20 acres of this land[34]. Current Use involves a lien that is attached to the deed of the property; if the landowner removes the lien on their deed and withdraws their land from the program, they must pay a land use change tax that is 10% of the full fair market value of the land[34]. While carbon credit programs and carbon offset markets tend to have much more stringent requirements than the Current Use Program, they typically require a management plan, something that the Current Use program already requires. Both Current Use and carbon offset programs include a withdrawal penalty as well. While the Current Use Program focuses on active timber management and carbon credit programs focus on bolstering carbon stocks, the withdrawal penalty and forest management plan requirements of the Current Use program match some of the most fundamental aspects of carbon credit programs. Furthermore, Current Use landowners agree to put their land into Current Use in perpetuity, as the lien runs with the land even when it is sold. This focus on long term management aligns with the long contract length of carbon credit programs, when land must be managed to store carbon for 20–100 years, depending on the nature of the specific program[35]. Overall, by focusing on Current Use forest landowners in Vermont, we highlight a population that is likely to be familiar with many concepts surrounding land management that are complementary to carbon offset programs. Thus, this population may provide key insights into how to integrate small forest landowners into larger forest carbon offset programs.

### Best worst scaling (BWS) and best worst choice (BWC)

This study implements an innovation in BWS, called BWC, to assess landowner preferences for carbon credit programmatic features—from two distinct dimensions of utility. BWC is a hybrid survey method, which enables estimates of BWS (e.g.,[33],[36]) and DCE (i.e., Binary Choice Method; [37]) using the following two tasks: 1) BWS task, choose a ‘best’ and a ‘worst’ item (attribute level; i.e., carbon credit programmatic feature) from a given list of items (choice profile; i.e., carbon credit program); and 2) DCE task, choose to ‘accept’ or ‘reject’ the entire profile as a whole. BWS was originally designed to overcome some of the limitations of traditional DCE[38]. Some DCEs do not allow for direct comparisons/tradeoffs amongst preferences and conjoint measurement techniques that employ rating scales, such as a 1–10 or 1–100 scales, to assess direct tradeoffs fail to account for the introduction of inherent human biases with subjective rating scales (e.g., your ‘5’ might be another person’s ‘3’ on a rating scale, or vice versa[38]). BWS is grounded in random utility theory and eliminates the need to make unnecessary assumptions on how respondents answer questions using ratings scales or rankings [39]. BWS methods place all the attributes on an underlying utility scale for a given respondent as they are asked to choose a best and worst feature of each choice profile[39]. The BWC technique is particularly applicable in situations where the choice is unfamiliar to the respondents and they are not experienced consumers of the product or service they are being asked to assess [40]. Landowners tend to be unfamiliar with carbon credit programs ([16]), further prompting us to utilize BWC instead of a more traditional conjoint measurement technique for this study.

Furthermore, unlike traditional DCEs, the BWS component of BWC will obtain estimates of both the importance of a given attribute as-a-whole as well as the unconditional demand utility associated with a given level of an attribute[39]. While other feasibility studies look at landowners’ WTA compensation for engaging in carbon credit programs, BWC allows us to understand the nuances of landowner preferences. We are also able to understand how different demographic groups vary in their preferences for different attributes. BWC has only recently been applied to natural resource management cases (e.g.,[14]). Overall, this study seeks to contribute to the literature that applies BWC to the field of natural resource management, as well as the literature on small forest landowner preferences for carbon credit program attributes.

### Survey design

Our survey sought to obtain landowner willingness-to-accept (WTA) compensation for participating in a carbon credit program and, thus, produce carbon offsets[14]. To do so, our survey design focused on four key features (or attributes) of carbon credit programs: the organization in charge of implementing the program, the estimated revenue to the landowner, the minimum program duration (otherwise known as contract length), and the magnitude of withdrawal penalty from program. We used a snowballing, semi-structured interview process, a review of current carbon offset protocols and markets (especially California CARB’s program), and a literature review to finalize this list of attributes and develop realistic levels of each attribute in the context of the state of Vermont (see Table 1). In addition to questions about carbon credit programs, we also asked landowners to fill out information about their demographics, forest management practices, and views regarding climate change.

**Table 1:** Attributes and levels used to create hypothetical carbon credit programs.

Attribute	Definition	Levels
Organization	Organization that implements carbon credit program	For-profit company
		Non-profit company
		Government organization
Revenue	Per-acre, per year payment from program, after costs	$5
		$10
		$15
Program Duration	Commitment length to the program	20 years
		60 years
		100 years
Withdrawal Penalty	Per-acre fine for leaving the program early	$0 (no penalty)
		$50
		$100

Following the methods of several recent studies, we included varying levels of potential revenue in our surveys to be able to obtain WTA estimates for other variables of the program[9,12,14]. We selected three potential revenue levels for Vermont carbon credit programs: $5, $10, and $15 per acre per year, levels which were determined by modelling a variety of revenue scenarios across program durations for the Northeast region (See Table 2). We chose 20, 60, and 100-year program durations to represent the range of contract length requirements for carbon offset market programs in the United States: 20-year minimum commitment for the Verified Carbon Standard (VCS) to a 100-year commitment for CARB’s program[18]. Table 2 demonstrates the modelling exercise used to calculate potential per acre, per year revenue for carbon credit programs in the Northeast. The calculations take into account two possible upfront payments for the carbon credit program in the first year: a high of $250 per acre and a low of $100 per acre[41]. Then, for each subsequent year, we consider two possible per acre per year payments: a high of $10 per acre per year and a low of $5 per acre per year[41]. The two medium revenue scenarios consider a high upfront payment and a low per year payment, or vice versa. The combinations of high and low upfront and yearly payments across contract lengths show a range of approximately $5 to $15 in per acre per year payments, when averaged across the entire project duration. We conducted this analysis in order to ensure that the revenue levels in our hypothetical carbon credit programs are as realistic as possible. We also considered three withdrawal penalty levels instead of just the presence of a penalty or not[10,14]. Finally, we distinguished between non-profit, for-profit, and government implementing entities as potential program implementers.

**Table 2:** Selection of potential average, per acre, per year revenue generation scenarios.

	20-year Contract	60-year Contract	100-year Contract	Average
High Revenue	$\frac{1(250)+19\;(10)}{20}=\frac{\$22.00}{acre-year}$	$\frac{1(250)+59\;(10)}{60}=\frac{\$14.00}{acre-year}$	$\frac{1(250)+99\;(10)}{100}=\frac{\$12.40}{acre-year}$	$\frac{\$16.13}{acre-year}$
Medium Revenue	$\frac{1(250)+19\;(5)}{20}=\frac{\$17.25}{acre-year}$	$\frac{1(250)+59\;(5)}{60}=\frac{\$9.08}{acre-year}$	$\frac{1(250)+99\;(5)}{100}=\frac{\$7.45}{acre-year}$	$\frac{\$11.26}{acre-year}$
Medium Revenue	$\frac{1(100)+19\;(10)}{20}=\frac{\$14.50}{acre-year}$	$\frac{1(100)+59\;(10)}{60}=\frac{\$11.50}{acre-year}$	$\frac{1(100)+99\;(10)}{100}=\frac{\$10.90}{acre-year}$	$\frac{\$12.30}{acre-year}$
Low Revenue	$\frac{1(100)+19\;(5)}{20}=\frac{\$9.75}{acre-year}$	$\frac{1(100)+59\;(5)}{60}=\frac{\$6.58}{acre-year}$	$\frac{1(100)+99\;(5)}{100}=\frac{\$5.95}{acre-year}$	$\frac{\$7.43}{acre-year}$

**Fig 1:**
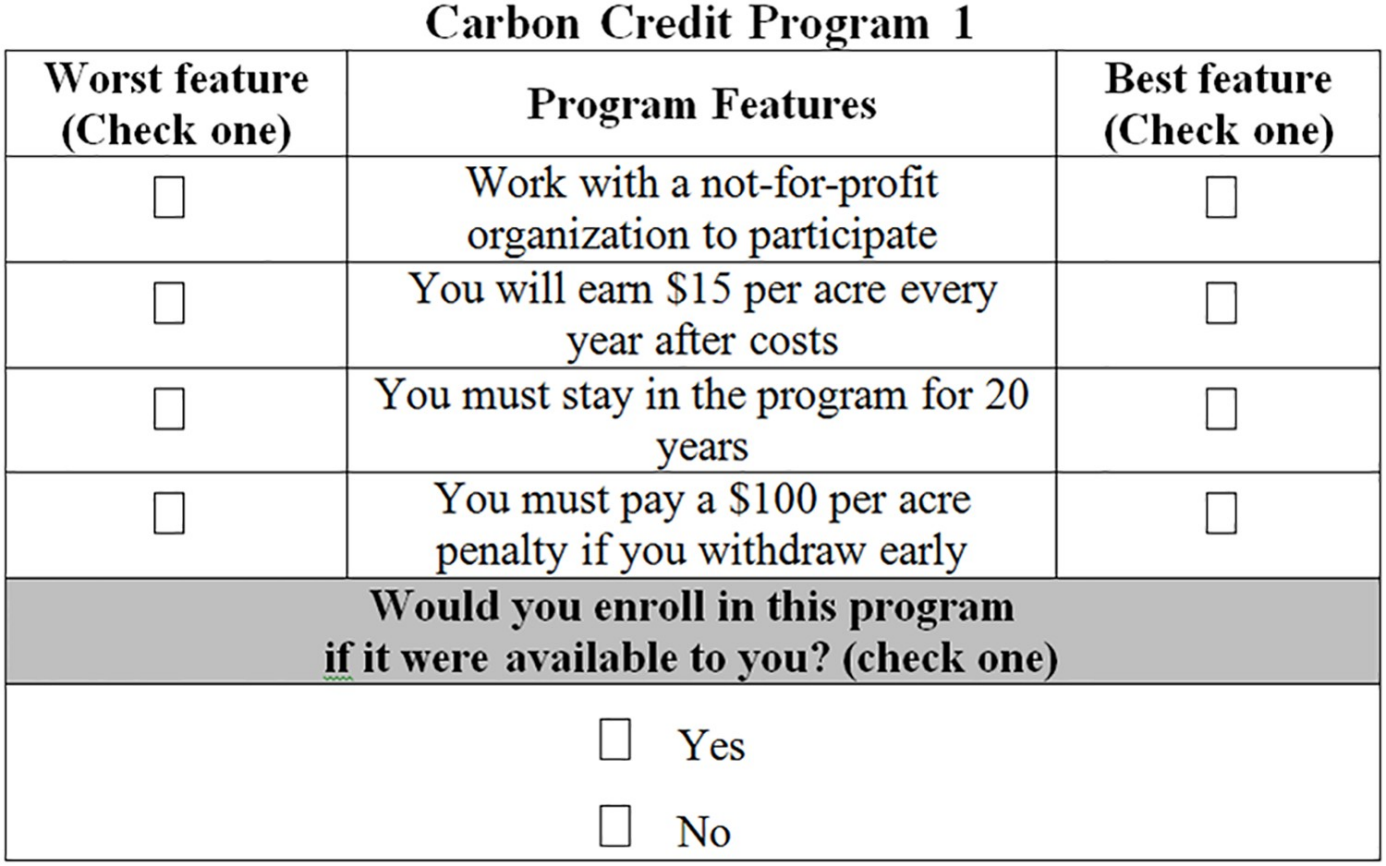
Choice scenario presented to respondents in mail survey.

Given the number of variables in Table 1, there were 81 possible combinations (3^4^; 4 attributes with 3 levels each) of hypothetical carbon credit programs that include each attribute once with one specific level of the attribute. In order to keep the survey to a reasonable length for respondents, we utilized an orthogonal main effects plan (OMEP) to construct nine choice scenarios for hypothetical carbon credit programs[42]. Each attribute has three possible levels, allowing our OMEP to be symmetric and balanced—the latter recommended by Flynn et al. (2007)[38]. This design helps reduce standard errors for parameter estimates, compared to an imbalanced design where different attributes have different numbers of levels[42]. These nine choice scenarios, also known as “profiles”, were designed following Soto *et al*. (2016) as well as Louviere *et al*. (2000) [14][36]. Fig 1 shows the first of the nine choice scenarios presented to respondents. For each forest carbon credit program, respondents were asked to choose a ‘best’ (most preferred) feature and a ‘worst’ (least preferred) feature, the BWS task, and also to consider the entire forest carbon credit program and whether they would enroll in it or not if given the option, the DCE task. For ease of the reader, we hereafter refer to the selection of best and worst attributes, the BWS task, as “Task 1” and the decision to enroll in the program, the DCE task, as “Task 2”. Respondents were specifically instructed to consider each profile separately and not compare a given profile to other profiles when performing Task 2. Task 1 allows for the direct comparison of the utility of different attributes and attribute levels using BWS [38], whereas Task 2 assesses utility ‘indirectly’ by making inferences on specific attribute levels from decisions on entire choice profiles. Note that Task 1 also produces conditional demand data [32], which requires respondents to choose two items, whereas Task 2 is consistent with traditional economic demand theory (unconditional demand, which allows for respondents to opt-out from choosing anything [43]).

In the survey, respondents were asked to read a list of frequently asked questions (FAQ) about carbon credit programs before filling out their preferences for each carbon credit program. We anticipated that many survey respondents would be unfamiliar with the features of carbon credit programs as well as the direct and opportunity costs to participating in a carbon credit program, so we elucidated these details in the FAQ. Every respondent received the same survey containing four sections: questions about forest management and ownership, questions about their views regarding climate change, the nine hypothetical carbon credit programs (including FAQ), and questions regarding their social demographics. In total, the survey included nine hypothetical carbon credit scenarios and 24 additional questions. We include the full survey and related survey implementation materials in the Appendices of this manuscript. (See S1 to S4 Appendices).

### Econometric analysis

For Task 1, we employed the random utility framework (RUF) of BWS to understand how often one attribute is picked over another and to model the real world context of decision-making and tradeoffs [40]. With respect to Task 2, we employed a binary choice model, also rooted in RUF, which enabled the estimation of WTA for programs [37, 39]. Task 1 was estimated using a conditional logit model to obtain attribute impact and level-scale values and for Task 2 we used a random effects logit (REL) model [10,14,38,43]. We obtained estimates for the second task with all variables’ effects coded and with revenue quantitatively coded. We refer to these as Models 1 and 2, respectively. The latter coding allowed us to obtain WTA estimates in dollars per acre per year for the attributes of the carbon credit program other than revenue.

### Conditional logit (BWS)

We used a paired model analysis to analyze the BWS data rather than marginal model analysis in order to avoid the potentially large standard errors associated with the latter [38]. The conditional logit model is an extension of a multinomial logit model described by [33], where *P*_*ij*_|*C* is the probability of choosing *i* as the best attribute level and *j* as the worst attribute level in a given profile *C*. *δ*_*ij*_ + ϵ_*ij*_ is distance between *i* and *j* on an latent scale of utility including a random disturbance term and *Max*(*δ*_*kl*_ + ϵ_*kl*_) is the largest of all other paired difference in *C* [33]. Assuming IID Gumbel distribution of ϵ_*ij*_, this multinomial logit model can be expressed as:

$$P_{ij}|C=\text{exp}\left(\delta_{ij}\right)/\sum_{kl\in C}\text{exp}\left(\delta_{kl}\right)$$ (1)

The conditional logit model was used to assess individual best-worst pair choices, conditioned on all best-worst pairs available for a given program (choice profile) [14,38]. We used Stata 14 software to estimate the parameters in Eq (1). As such, we expanded each carbon credit program for each respondent into the number of possible best-worst pairs that could be chosen for each program in order to be able to use the conditional logit Stata command for a paired model [38]. Each carbon credit program would have J(J-1) possible best worst combinations, where J is the number of attributes in each program, in this case, four. This led to 12 possible best-worst pairs that could be chosen per program. In Stata 14, observations for each respondent were stacked, generating nine observations per person, and then expanded by 12 to accommodate all potential best-worst pair options. The dependent variable, best-worst pair choices, was coded as 1 if chosen and 0 otherwise [14]. We utilize the following model, adapted from Soto *et al*. (2016) and Louviere *et al*. (2000) [38] [14]:

$$U_{diff}^{i}=\sum_{j=1}^{n}\beta_{j}^{i}D_{j}^{i}+\sum_{j=1}^{n}\sum_{k=1}^{m}\beta_{jk}^{i}D_{jk}^{i}+\varepsilon^{i}$$ (2)

The outcome of this equation, $U_{diff}^{i}$, is the difference in utility of each best-worst combination for the given carbon credit program. This equation includes the 12 possible best-worst combinations, *i* = 1, 2, … 12, where *n* is the total number of attributes, *j* indexes each attribute, while *k* indexes individual attribute levels, and *m* represents the total number of attribute levels of an attribute. Each attribute has an *attribute impact variable*, *$\beta_{j}^{i}$*, and each attribute level has a *level scale value*, $\beta_{jk}^{i}$, associated with it, allowing for the estimation of attribute impact (mean utility across all levels of an attribute) and the impact of each specific level scale value (deviations from mean utility), respectively [38]. $D_{j}^{i}$ is a variable that takes on a value of 1 if any level of attribute *j* is chosen as the best, -1 if *j* is chosen as worst, and 0 otherwise. Similarly, $D_{jk}^{i}$ is a variable that takes 1 if attribute level k is chosen as best, -1 if worst, and 0 otherwise. We opted to use effects coding rather than dummy variable coding in order to ensure that all attribute levels were centered on the mean attribute impact. In effects coding, one attribute level, from each attribute, is not explicitly included in the model but embedded in the other levels of the attribute[14]. The convention is also to omit the attribute impact variable with the lowest impact on utility (although any attribute impact and attribute level could be used) to avoid the “dummy variable trap” and to be used as the “reference case” [14,37,38]. This reference case takes on a value of zero on the latent scale of utility. Each omitted effects coded attribute level can be recovered as the negative sum of the other level-scale variables (Table 3). The lowest level of each attribute (for-profit company, $5 per acre per year, 20-year program duration, and no withdrawal penalty) was omitted from the regressions and “reverse coded” into the remaining two levels. Table 3 shows how the levels shown in column two and three are coded if the level in column 1 is chosen as the best feature. For example, if a for-profit company was chosen as best, the non-profit and government level scale values would both be coded as -1.

**Table 3:** Effects coding for analysis of best-worst scaling data.

Attribute	Effects Coding	Effects Coding
Organization	Non-profit organization	Government organization
For-profit company	-1	-1
Non-profit organization	1	0
Government organization	0	1
Revenue	$10 per acre, per year	$15 per acre, per year
$5 per acre, per year	-1	-1
$10 per acre, per year	1	0
$15 per acre, per year	0	1
Program Duration	60 years	100 years
20 years	-1	-1
60 years	1	0
100 years	0	1
Withdrawal Penalty	$50 per acre	$100 per acre
$0 per acre	-1	-1
$50 per acre	1	0
$100 per acre	0	1

After completing the effects coding of program characteristics, we generated interaction terms for each attribute impact with respondent level characteristics. Interaction terms were generated by multiplying each attribute impact by a covariate such as income, age, or education. These interaction terms were generated because respondent covariates did not vary across the 12 best-worst choices [44], and thus interaction terms were necessary to understand any heterogeneity within the survey population with regard to best and worst program features. The demographic terms in the final model were chosen following a process established in Flynn *et al*.[44]. We tested age, education, income, and gender as demographic variables and forest acreage and concern for climate change as respondent characteristics. All had at least one statistically significant impact on landowner carbon credit program preferences and were thus retained in our final regression[44].

The full model without interaction terms was estimated to identify the attribute impact with the lowest overall impact on respondent utility. We found duration of the program to be the least impactful attribute and was thus omitted from the final model so that it could be used as the reference case. Duration of the program was assigned a zero value such that all other attribute impacts could be interpreted on a latent scale of utility. The coefficient of attribute impacts in the conditional logit regression could then be interpreted as ranking the variables in order of importance, from the duration of the program variable with zero value to the variable with the highest attribute impact as the most important to overall utility.

### Random effects logit and willingness-to-accept (WTA)

In complement to Task 1, we utilized a random effects logit model to calculate WTA various characteristics of the carbon credit program. The random effects logit model was chosen to account for heterogeneity in responses for individual respondents due to socioeconomic or demographic factors or fatigue in completing the survey [14,43]. Random effects logit accounts for the unobserved heterogeneity that is typically a violation of assumptions behind DCEs [37,45]. By using the random effects logit model, we made the assumption that our observations were Bernoulli distributed[14,46]. Following Soto *et al*. (2016), we ran a random effects logit regression including only the level scale values as well as various quantitatively coded variables as independent variables[14]. Revenue was quantitatively coded in Model 2 rather than effects coded to allow us to obtain additional estimates of marginal WTA [14]. The WTA estimates obtained in Task 2 were marginal estimates, that is, the ratio of the attribute's marginal effects coefficient to the price coefficient, with units of United States (U.S.) dollars per choice. For each model, these estimates were specified as the ratio of marginal effect of the level scale value to the quantitatively-coded revenue coefficient in U.S. dollars per acre per year.

### Sampling and survey implementation

After the survey was designed, it was approved in writing through the Dartmouth RAPPORT research portal by Dartmouth College’s Committee for the Protection of Human Subjects (CPHS), which serves as the Institutional Review Board (IRB) for Dartmouth College. In order to choose the survey sample, we took a stratified random sample of all forest landowners in Vermont’s Current Use program. The sample was taken from a publicly available database provided by the Vermont Department of Taxes in September 2016. After consolidating owners with multiple entries in the database and removing landowners who did not own forest land, we stratified forest landowners into three categories: landowners with less than 100 acres, landowners with 100–200 acres, and landowners with over 200 acres. The three strata were thus allocated according to their proportional representation in the survey sample: 640 surveys for the less than 100 acres stratum, 219 surveys for the 100–200 acres stratum, and 133 surveys for the 200+ acres stratum. This stratification was done to ensure that different landholding sizes were adequately represented in the survey and to ensure that large landowners were not overrepresented in the sample.

The Vermont Current Use program had a population of approximately 12,340 landowners in 2015. In order to remain within a margin of error of 5% for a population of this size, we were seeking to receive approximately 400 completed surveys. Given the 21–51% response rate of comparable surveys, from January through March of 2017, we administered 992 mail surveys with the hope of an approximately 40% response rate [10,12,14,15]. In order to obtain the highest response rate possible, we implemented a mail survey following the widely used Dillman Tailored Design Method [47] [10,12]. All Dillman Tailored Design Method materials that were sent to landowners can be found in the Appendices (S1 to S4 Appendices). Survey participants did not give formal written or oral consent to participate, but survey response was entirely voluntary by the participant. In the cover letter of the survey mailing, participants were informed that their responses would be maintained anonymously if they chose to respond to the survey.

## Results and discussion

### Summary statistics

Out of 992 surveys sent out to Vermont forest landowners, 481 surveys were returned for an overall response rate of 48.4%. Of the 992 surveys, 53 were undeliverable due to old or flawed address information. Of the 481 surveys returned, 233 surveys fully completed both tasks and answered all relevant demographic questions included in the interaction terms in the analysis. Of the 248 surveys that were returned incomplete, 28 were return completely blank, 37 were missing key demographic information, and the remaining 183 did not fully complete every best-worst choice profile. Unlike DCEs which just ask a respondent to accept or reject a profile, we also ask respondents to choose the best and worst feature of that program before they chose to accept or reject it. These additional tasks may have led to greater fatigue for respondents, leading them to not fully complete the survey. Still, the total response rate and the fully completed survey response rate were consistent with the 20–51% response rate for similar surveys of forest landowners[10,12,14]. Given the high rate of incompleteness in the surveys, we analyzed the differences between those landowners who fully completed the survey and those who did not. Among the landowners who did not fully complete their surveys, there are a greater number of older landowners, lesser-educated landowners, lower income landowners, and female landowners. However, performing difference in means tests for each of these demographic variables showed that the landowners who fully completed the survey versus those who did not fully complete surveys are not statistically significantly different on average. As a whole, our final data set of 233 fully completed surveys may reflect the preferences of Vermont Current Use forest landowners who are more concerned with carbon credit programs and were thus willing to complete the whole survey. Of the 233 fully completed surveys, the average landowner in the survey owns their forest jointly and owns a median of 85 acres of land held in one parcel that is not fragmented. The median date of land purchase or acquisition is 1998. Unless otherwise specified, the following summary statistics represent the 233 respondents who fully completed the survey and were included in our analysis.

### Respondent statistics

**Fig 2:**
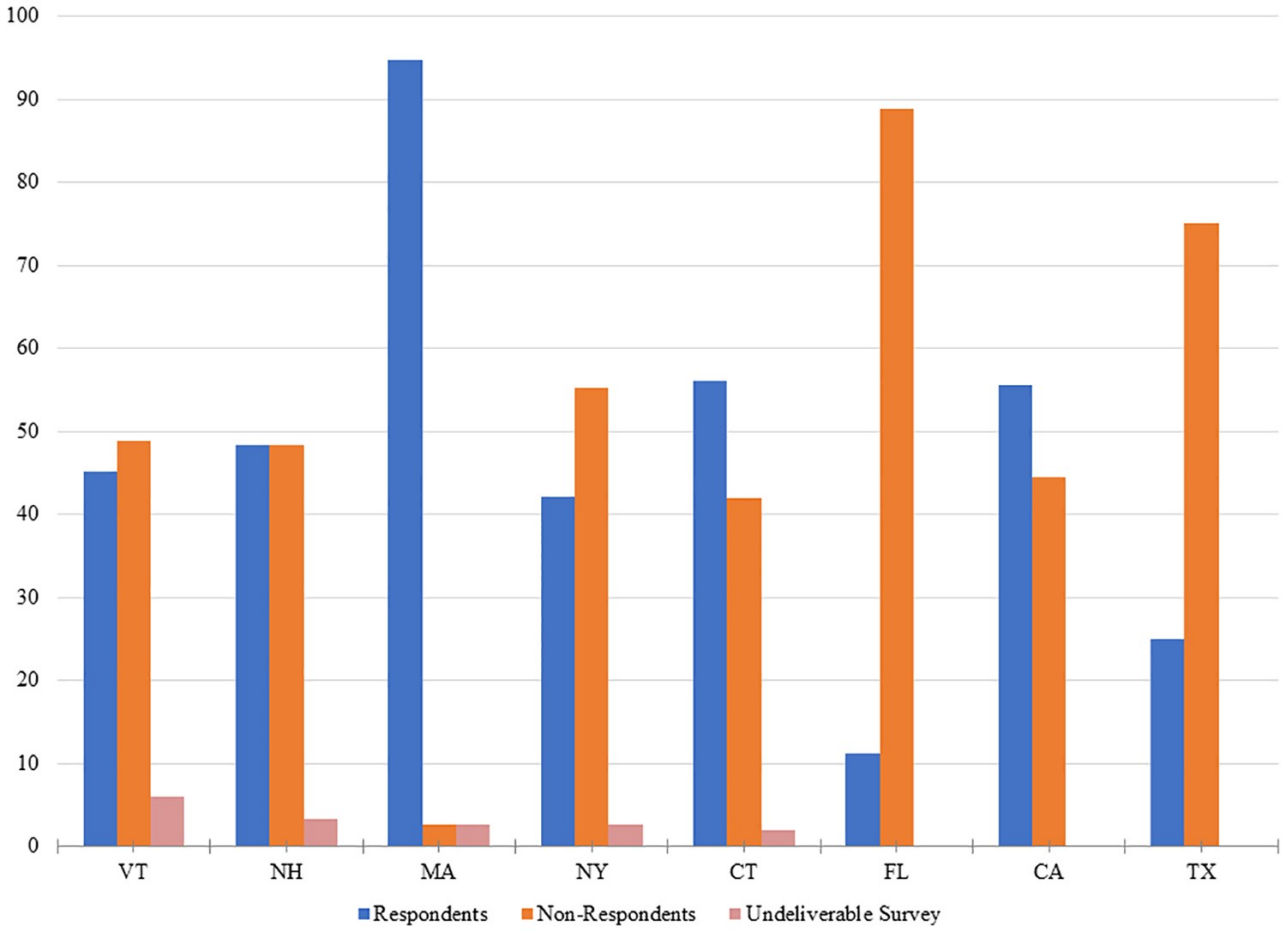
Percent respondents, non-respondents, and undeliverable surveys by state of residence of landowner in survey sample.

In order to address potential non-response bias, we analyzed both the respondent and non-respondent groups based on available demographic and landholding information. As shown in Fig 2, there was little potential for bias due to fewer out-of-state respondents. While Vermont landowners living in Massachusetts and California were more likely to respond, Florida and Texas residents were less likely. As a whole, out of state landowners did not systematically have lower response rates. As another test of responsiveness, we checked in our three survey strata to compare response and non-response rates in each stratum. In all three strata, non-response rates were slightly higher than response rates but were consistent across strata. The response rate for the three strata (less than 100 acres, 100–200 acres of land, greater than 200 acres of land), were 50.1%, 53.4%, and 54.9%, respectively. We conducted a chi-squared test to examine if the difference in response rates between the three groups is statistically significant and, with a chi-squared value of 1.39 and a sample of 993 observations, we did not find significant differences in response rates between groups. This suggests that landowners of all landholding sizes were equally likely to respond to the survey and that the survey is not biased toward or over representative of one group of landowners.

Overall, respondents owned an average of 170 acres of land while non-respondents owned only 118 acres of land on average. However, we found that the average landholding size for respondents was skewed due to several large landholdings in the portion of the sample that did respond to the survey. To better understand potential non-response bias on forest acreage, the four largest landowners were removed from the average acreage calculations. After removing these landowners from the calculation of average parcel size, non-respondents and respondents show negligible difference in the average number of forest acres owned. On average, after removing these outliers, respondents own 113 acres of forest land while non-respondents own 115 acres of forest land. As a whole, this analysis suggests that respondents are not categorically different than non-respondents.

### Conditional logit

In our conditional logit analysis, all of the attribute and level-scale values were significant to the 1% level except for the level scale value of receiving $10 per acre per year of revenue and the level scale value of a 60-year program duration (Table 4). For the attribute impacts, revenue was the most preferred attribute. After revenue, withdrawal penalty was the second most valued or important attribute, followed by organization type. Program duration was the least valued attribute. For the level-scale values, the coefficient for non-profit organization was positive and significant, .992 (0.393) while the coefficient for government organization was negative and significant, -0.801 (0.387). This indicates that landowners strongly preferred forest carbon credit programs to be implemented by non-profit organizations over for-profit and government entities. Landowners strongly preferred the highest level of compensation, $15 per acre per year revenue from the program and the lowest level of commitment to the program, the 20-year program duration. However, it is worth noting that the difference in the magnitude of the coefficients between the 60-year and 100-year program duration was much less than the difference between the 20 and 60-year durations. Landowners strongly preferred that there be no withdrawal penalty from the program. However, if there was a withdrawal penalty, landowners appeared to very slightly prefer a $100 per acre withdrawal penalty, with a coefficient of -0.929 (0.374) over a $50 per acre withdrawal penalty, with a coefficient of -1.064 (0.377). Given that the two coefficients are negative on the latent utility scale and close in magnitude, respondents viewed both $50 and $100 per acre withdrawal penalties as similarly unfavorable to them and may not strongly prefer one penalty over the other.

**Table 4:** Conditional logit level-scale impacts.

Level-Scale Impacts	Coefficient	Standard Error
for-profit organization	-.191	
non-profit organization	0.992**	(0.393)
government organization	-0.801**	(0.387)
5 dollars per acre, per year	-1.21	
10 dollars per acre, per year	-0.00920	(0.439)
15 dollars per acre, per year	1.220***	(0.466)
20-year program duration	1.192	
60-year program duration	-0.362	(0.383)
100-year program duration	-0.830**	(0.402)
no withdrawal penalty	1.993	
50 dollars per acre withdrawal penalty	-1.064***	(0.377)
100 dollars per acre withdrawal penalty	-0.929**	(0.374)
**Observations**	**23,328**	

Standard errors in parentheses,

*** p<0.01,

** p<0.05,

* p<0.1

After analyzing the respondents as one group, we sought to understand how different demographic groups within the respondent pool responded to carbon credit programs (Table 5). The interaction terms we added to test this were age, gender, education level, income, total forest acreage, whether or not landowner is a timber harvester, and whether or not landowner is concerned about climate change. In this study, we define a timber harvester as someone who has sold timber from his/her forest in the past five years. As shown in Table 5, demographic groups did show significant differences as to the additional or diminished impact of the attribute as a whole. Older landowners tended to see revenue and withdrawal penalty as slightly less important to the program with coefficients of -0.191 (0.0597) and -0.0790 (0.0427), respectively. Landowners that are concerned about climate change also saw revenue and withdrawal penalty as significantly less important with coefficients of -0.442 (0.218) and -0.293 (0.154), respectively, while organization implementing the program was significantly more important with a coefficient of 0.463 (0.160). Timber harvesters also considered the revenue aspect of carbon credit programs less impactful as an attribute overall with a differential attribute impact of -0.526 (0.148). The results for these demographic groups suggest different preferences with regard to carbon credit programs. Timber harvesters may not be as concerned about revenue from carbon credit programs because they already generate revenue from timber harvests, which they can continue to do in some capacity as part of a carbon credit program. On the other hand, those that are worried about climate change may not care as much if they make revenue or have to pay a withdrawal penalty for the program because they support the idea of forest carbon credits on principle or are possibly less concerned with monetary aspects of the program.

**Table 5:** Conditional logit attribute impacts.

Attribute Impacts	Coefficient	Standard Error
duration of program	0 (reference variable)	
Organization	1.507***	(0.350)
Revenue	4.875***	(0.440)
withdrawal penalty	1.596***	(0.314)
age*organization	-0.0111	(0.0477)
age*revenue	-0.191***	(0.0597)
age*withdrawal penalty	-0.0790*	(0.0427)
female*organization	-0.381***	(0.134)
female*revenue	-0.525***	(0.162)
female*withdrawal penalty	-0.0378	(0.122)
education*organization	-0.184***	(0.0532)
education*revenue	-0.0915	(0.0648)
education*withdrawal penalty	-0.0950**	(0.0465)
income*organization	0.143***	(0.0456)
income*revenue	0.0229	(0.0554)
income*withdrawal penalty	0.111***	(0.0405)
acres*organization	9.18e-05	(8.10e-05)
acres*revenue	0.000783**	(0.000343)
acres*withdrawal penalty	8.40e-05	(5.76e-05)
timber harvester*organization	-0.198*	(0.121)
timber harvester*revenue	-0.526***	(0.148)
timber harvester*withdrawal penalty	-0.144	(0.107)
climate change concern*organization	0.463***	(0.160)
climate change concern*revenue	-0.442**	(0.218)
climate change concern*withdrawal penalty	-0.293*	(0.154)
**Observations**	**23,328**	

Standard errors in parentheses,

*** p<0.01,

** p<0.05,

* p<0.1

In the “Attribute Impacts” column, * indicates an interaction between the two variables

### Task two: Random effects logit and willingness-to-accept (WTA)

For Task Two, we were able to quantify the willingness of forest landowners to accept various aspects of the program. As shown in Table 6, the shortest program duration, 20 years, was positive while 60 and 100-year program durations had negative and significant coefficients. In addition, no withdrawal penalty was positive while $50 per acre and $100 per acre withdrawal penalties were negative and significant. While the 100-year program duration was consistently more negative and larger in magnitude than the 60-year program duration coefficient, the $100 per acre withdrawal penalty coefficient and the $50 per acre withdrawal penalty were very similar in magnitude. Similar to the interpretation from Task 1, $50 and $100 per acre penalties tended to be viewed similarly by respondents and were less preferred than no withdrawal penalty. The results for organization type shed light on landowners’ views on governance of carbon credit programs. The coefficient for non-profit organization was positive and significant to the 1% level, with a value of 0.446 (0.095) while the coefficient for government organization was negative and significant, with a value of -0.161 (0.097). The coefficient for a for-profit company managing the carbon credit program is also negative, at -0.285. This suggests that having a for-profit company or government organization administer the program decreases landowner willingness to accept the program whereas having a non-profit organization administer increases willingness to accept.

**Table 6:** Results from binary-choice model: Random effects logit model.

	Model 1 All Effects Coded	Model 2 Revenue Quantitative	WTA Model 2 (USD per acre per year)
For-profit company	-0.279^b^	-0.285^b^	$1.88
Non-profit organization	0.445*** (0.095)	0.446*** (0.095)	($2.93)
Government organization	-0.166* (0.098)	-0.161* (0.097)	$1.06
Rquant		0.152*** (0.018)	
5 dollars per acre, per year	-0.788^b^		
10 dollars per acre, per year	0.0480 (0.093)		
15 dollars per acre, per year	0.740*** (0.096)		
20-year program duration	1.183^b^	1.179^b^	($7.76)
60-year program duration	-0.396*** (0.097)	-0.400*** (0.097)	$2.63
100-year program duration	-0.787*** (0.103)	-0.779*** (0.102)	$5.13
No withdrawal penalty	1.151^b^	1.148^b^	($7.55)
50 dollars per acre withdrawal penalty	-0.591*** (0.098)	-0.588*** (0.097)	$3.87
100 dollars per acre withdrawal penalty	-0.560*** (0.103)	-0.560*** (0.103)	$3.68
Constant	-0.996*** (0.192)	-2.513*** (0.271)	
Number of respondents	233	233	
Total number of choices	2,106	2,106	
Log-likelihood	-968.39	-968.52	
Chi-square statistic^a^	625.92	625.73	

Standard errors in parentheses,

*** p<0.01,

** p<0.05,

* p<0.1

^a^ Chi-square statistic of hypothesis test that all model parameters equal zero

^b^ Effects coded coefficients are the negative sum of the two other level-scale values of the attribute

**Fig 3:**
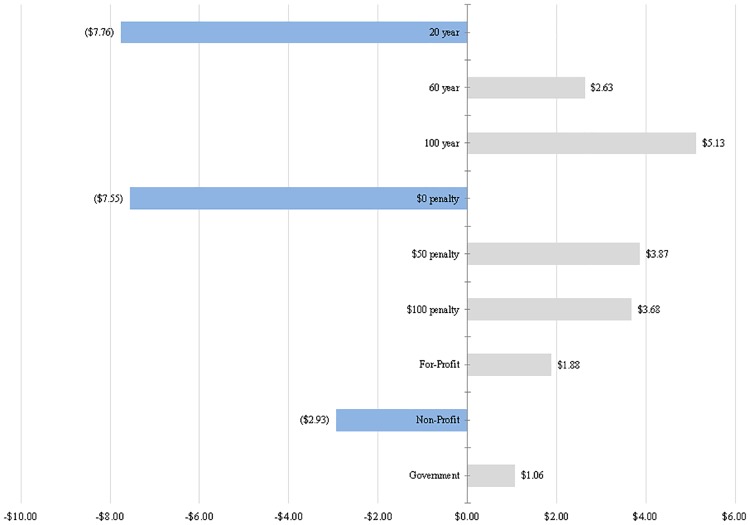
Willingness to accept carbon credit program feature from random effects logit model binary choice (USD per acre per year/choice).

In Model 2, wherein revenue was quantitatively coded, we generated a quantitative measure of landowner willingness to accept (WTA) carbon credit programs, shown in the last column of Table 6. This quantitative measure can be thought of as how much more or less the program would have to pay landowners in order for them to accept a given level of a given feature of the program. The WTA measure is calculated by dividing the level-scale coefficients in Model 2 by the Rquant variable in Model 2. For Model 2, to get landowners to accept a 100-year program duration required $5.13 per year per acre in compensation while a 60-year program duration required only $2.63 per year per acre. For both the $50 and $100 withdrawal penalties, a landowner would require approximately $3.87 or $3.68 in compensation costs per acre per year, respectively. To accept a government organization or for-profit company running the program, landowners desired $1.06 or $1.88 per acre per year in additional compensation, respectively. On the other hand, a 20-year program duration, with no withdrawal penalty, run by a non-profit organization, according to Model 2, would decrease compensation costs by $7.76, $7.55, and $2.93, respectively for each feature. These willingness-to-accept estimates from Model 2 are visualized in Fig 3. Negative values for WTA indicate a reduction in the amount a program would have to pay landowners to accept a setup with this feature while positive values indicate the additional compensation landowners would require to accept this feature of the program.

In addition, we sought to understand how much landowners would have to be compensated to switch between different levels of a given attribute. This amount was calculated in dollars and by computing the difference between WTA amounts calculated for two levels. Following the last column in Table 6, it would require $10.39 per acre per year in compensation to get a landowner to switch from a 20-year to a 60-year program duration. To switch from a 60-year to 100-year program duration would require only $2.50 per acre per year. Including a withdrawal penalty would necessitate a payment to the landowner of $11.42 per acre per year for them to switch from no penalty to a $50 per acre withdrawal penalty. In order to get a landowner to switch from a non-profit to a government organization as the implementer of a carbon credit program, the program would need to compensate the landowner an additional $3.99 per acre per year. As a whole, this suggests that getting landowners to accept withdrawal penalties and longer program durations is more costly than getting them to accept a different implementing organization.

The results of this study are relatively consistent with other studies on the preferences of forest landowners for carbon market participation [10–12,14,47]. Our findings are most directly comparable to Soto *et al*. [14] because both studies utilized BWC methods. While preference elicitation techniques used in other studies do not provide the same kind of WTA estimates that we found in this study, their results suggest that participation rates in carbon credit programs increase as per acre per year revenue increases and decrease as contract lengths increase, which is consistent with our results [10,11]. However, the estimates by Markowski-Lindsay *et al* (2011) do not reflect the same range of potential program revenues as this study as they used $10, $100, and $1000 per acre per year revenue amounts[10]. Moreover, those estimates focused on willingness to participate in three programs and found very low participation rates in all three programs[10]. In our study, we found that 14–60% of the sample that fully completed the survey would participate in a carbon credit program, depending on the program in question.

Willingness-to-accept carbon credit programs can be measured at the program level or the individual attribute level. At the program level, Miller *et al* (2012) found that payment or revenue and withdrawal penalty were both significant predictors of program participation and that $18 per year per acre would be required for 50% participation in the program[12]. Rather than generate WTA estimates at the program level, we generated results similar to those by Simpson and Li (2011) and Soto *et al*. [31][14]. The former found that Texas landowners required $19.92 per acre per year to accept a five-year contract and $27.36 to accept a conservation easement status [36]. Similarly, Florida landowners required $28.53 per acre per year more in compensation to accept a 100-year commitment to the program and $15.42 per acre per year more in compensation to accept a 40-year commitment to the program [14]. Our results showed that Vermont forest landowners in the Current Use program required much lower compensation per acre per year to accept similar program features. Our study found that for a 60 or 100-year program duration, landowners were willing to accept these features for only $2.63 or $5.13 per acre per year in additional compensation, respectively. This suggests that Vermont could be a more viable location in which to pilot carbon credit programs under current carbon offset market conditions and potential revenue levels. Furthermore, landowners may be more willing to accept a government or for-profit program implementer if doing so would mean a shorter contract length.

Ours is the first study on willingness to accept forest carbon credit program in the state of Vermont and only the second study to employ BWC to understand forest landowner preferences for carbon credit programs. In addition, this study allowed us to glean how different demographic groups within the Vermont Current Use forest landowner population respond to carbon credit programs. While Soto *et al*. did not find significant differences between demographic groups[14], we found significant heterogeneity in preferences. For one, older, female, timber harvesting, and climate change-concerned landowners do not appear to consider revenue as important of an attribute on the underlying utility scale of all attributes of the program. For organization type, more highly-educated female landowners care less about the implementing organization while landowners concerned about climate change care significantly more about the organization type. This information could give policy makers a sense of how to design carbon credit programs to appeal to different demographic groups within the larger population of Vermont Current Use forest landowners. While these results only speak to our unique survey population, further research could show whether our findings are generalizable to other states, particularly those with forest landowner programs similar to Current Use, and other populations of forest landowners.

### Methodological advances of best-worst choice

This study helps further explore the method of BWC and its applications in environmental and natural resource management settings. Our use of both BWS and a dichotomous choice for each carbon credit program allowed us to measure the indirect and direct utility of carbon credit programmatic features. This is a distinct advantage over traditional DCEs that ask respondents to choose one carbon credit program over another [9–11]. Overall, we contribute to the BWC literature surrounding carbon credit programs by examining new programmatic variables and applying BWC techniques in the context of the state of Vermont. Our results also detail which carbon credit program attributes are more important to certain demographic groups. This helps stakeholders understand how different demographic groups in Vermont perceive carbon credit programs and their features.

### Policy implications

Overall, our results suggest several potential policy options for carbon credit programs for small forest landowners. First and foremost, this study shows that Vermont may be a viable location to pilot carbon credit programs for small forest landowners. As discussed above, forest landowners in our survey population were willing to accept carbon credit programs for less compensation than landowners in Florida. Furthermore, our results suggest that forest landowners prefer that a non-profit organization, such as a local land trust or forestry organization, administer the aggregation of property for a carbon offset project. Having a non-profit organization implement the program reduces the compensation landowners require by $2.93 per acre per year. Carbon credit programs could potentially manifest themselves as easements, agreements between landowners and non-profit land trusts in order to manage the land for carbon storage over at least a 100-year time period to meet CARB requirements[48].

Our findings further corroborate the idea that revenue is an important factor in forest management decisions for small forest landowners[16,17]. However, our data also suggest that carbon offset projects in the Northeast region of the United States could potentially generate enough revenue to convince small forest landowners to accept carbon credit programs. In the Northeast, carbon credit programs have the potential to generate a large amount of revenue upfront but only $5-$10 per acre per year following this initial period. This revenue, when averaged, could allow landowners to receive $5-$15 per acre per year from carbon credit programs, as included in this survey. The results shown in Table 6 suggest that this amount of revenue could be sufficient to facilitate landowner buy-in. For one, landowners only required $5.13 in additional compensation to accept CARB’s standard 100-year commitment to the program. Switching from a 20-year to a 100-year program duration would therefore only require a total of $12.89 per acre per year, within the potential revenue range for these programs. Moreover, this survey found that, depending on other program characteristics, up to 40% of landowners are willing to accept a program with a 100-year commitment. This suggests that carbon credit programs among the Current Use population in Vermont could garner sufficient landowner participation while meeting CARB requirements.

Since Current Use forest landowners already face a withdrawal penalty from the Current Use program, they may not require as much compensation to accept a withdrawal penalty from the carbon credit program. Even so, landowners only require $11.42 per acre per year to switch from not having a withdrawal penalty to having one. This compensation amount is also within the range of $5-$15 per acre per year potential revenue from forest carbon credit programs. From a compensation perspective, as long as the requirements for forest carbon credit programs are kept simple and as low cost as possible to small forest landowners, forest carbon credit programs could be viable for the Current Use population in Vermont.

While these findings indicate potential, in order to generate pilot forest carbon credit programs, several next steps must be taken to achieve a successful program for small forest landowners. As a first step, in order to engage small forest landowners in their compliance protocol, CARB would have to amend the Protocol to allow for aggregation of forest carbon offsets projects and to establish the process for obtaining a baseline for these projects. However, CARB will have to consider how to mitigate the risk of project reversal and the appropriate role of aggregators in a protocol that allows for aggregation. Second, further studies should be conducted to ensure that Vermont is a viable pilot location for forest carbon credit program from the supply side of carbon offsets. The level of carbon storage affects the most important feature of forest carbon credit programs for Vermont Current Use landowners: revenue per acre per year. Finally, further research should be conducted on the discounting of costs and benefits in forest carbon credit programs and on how small forest landowners respond to varying withdrawal penalties in forest carbon credit programs.

## Conclusions

In conclusion, forest carbon credit programs provide an opportunity to engage small forest landowners in the growing carbon offset market. These programs could provide revenue to small forest landowners as well as encourage long-term management of forests to sequester carbon as an alternative to clear-cutting and development. This study suggests that non-profit organizations should play a key role in forest carbon credit program implementation as respondents showed a statistically significant preference for non-profit program implementers. However, landowners are also willing to accept a different program implementer for less compensation than they require to switch to a longer project with a higher withdrawal penalty. Furthermore, aggregated carbon credit projects under an amended CARB protocol could be piloted in Vermont’s Current Use forest landowner population. This study shows that this population is willing to accept program requirements for reasonable levels of compensation given potential project revenue. In addition, this study adds to the literature on applications of BWC to natural resource management cases. As a whole, this study suggests that carbon offset project aggregation is a viable way to engage small forest landowners in the carbon offset market.

## Supporting information

S1 Appendix Pre-survey letter. Following Dillman Tailored Mail Survey guidelines, we sent out a letter to all landowners being surveyed to notify them that they would be receiving a survey.
(DOCX)

S2 Appendix Survey cover letter. (DOCX)

S3 Appendix Mail survey. (DOCX)

S4 Appendix Follow-up post card. Following Dillman Tailored Mail Survey guidelines, we sent a follow up post card to remind landowners to complete the survey after it had been mailed to them. We also sent every landowner a follow up mail survey, identical to that shown in S3 Appendix, two weeks after this post card.
(DOCX)
