# Supplementary material for: Small-scale forestry and carbon offset markets: An empirical study of Vermont Current Use forest landowner willingness to accept carbon credit programs
Source: PLoS One. 2018 Aug 14;13(8):e0201967. doi: 10.1371/journal.pone.0201967 (PMC6091951; doi:10.1371/journal.pone.0201967)
Supplement: S1 Appendix — Following Dillman Tailored Mail Survey guidelines, we sent out a letter to all landowners being surveyed to notify them that they would be receiving a survey. (DOCX) [file pone.0201967.s001.docx]

## S1 Appendix. Pre-Survey Letter


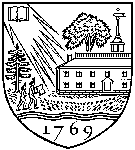
 Dartmouth College

|  |  | *Environmental Studies Program*  *6182 Steele Hall*  *Hanover, NH 03755-3571 USA* |
| --- | --- | --- |

January 9, 2017

Current Use Landowner

Address1

Address2

Town Zip

Dear Current Use Landowner,

I am writing to ask for your help with an important study being conducted for a senior honors thesis project. This study is being conducted under faculty supervision in the Environmental Studies Program at Dartmouth College, Hanover, NH, USA. A few days from now, you will receive a request in the mail to fill out a brief survey as part of this study. This survey concerns landowners like yourself who have forest land in Vermont’s Current Use Program and how you want to manage and generate income from your forest land in the future.

We would like to make it pleasant and easy for you to participate in this study. I am writing in advance because we have found that people appreciate knowing ahead of time that they will be asked to complete a survey for an academic study. You have been randomly selected out of all forest landowners in Vermont’s Current Use program to receive this survey and your participation is voluntary and confidential. By taking a few minutes to respond to the survey, you may help improve opportunities for forest landowners in Vermont’s Current Use program.

Thank you for your time and consideration. It is only with the generous help of people like you that our research can be a success.


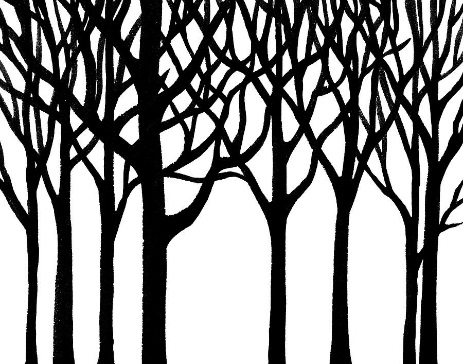


Best Wishes,

Alisa E. White

Dartmouth College Class of 2017

Environmental Studies Program

Senior Honors Thesis Candidate
