# Supplementary material for: Small-scale forestry and carbon offset markets: An empirical study of Vermont Current Use forest landowner willingness to accept carbon credit programs
Source: PLoS One. 2018 Aug 14;13(8):e0201967. doi: 10.1371/journal.pone.0201967 (PMC6091951; doi:10.1371/journal.pone.0201967)
Supplement: S2 Appendix — (DOCX) [file pone.0201967.s002.docx]

## S2 Appendix. Survey Cover Letter


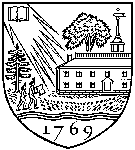
 Dartmouth College

|  |  | *Environmental Studies Program*  *6182 Steele Hall*  *Hanover, NH 03755-3571 USA* |
| --- | --- | --- |

January 12, 2017

Current Use Landowner

Address

Dear Current Use Landowner,

I am writing to ask for your participation in a survey being conducted as part of an honors thesis project, with supervision by a faculty advisor from Dartmouth College, Hanover, NH, USA. This important survey is part of a study about how forest landowners in Vermont’s Current Use program would like to manage and generate income from their forests in the future. By taking a few minutes to respond to this survey, you may help improve opportunities for forest landowners like yourself in Vermont’s Current Use program.

You are part of a **small group of forest landowners in Vermont’s Current Use program** that were chosen to participate in this survey. Your address was randomly selected and your participation is voluntary. Please make sure to have an adult from your household complete this survey. If your forest land is held in a business, estate, or trust, please have a representative or trustee take the survey. You may choose to not to respond to some questions. The survey should take about 15 minutes to complete. At the end of the survey, if you select that you are available for an interview, you may be contacted for a follow up interview. The results of the survey are available upon request.

The information collected in the survey will be **maintained confidentially.** Names and other identifying information will not be used in any presentation or paper written about this survey. If you have any questions about the survey, please contact Alisa White, the study director, by phone at (518) 222-3743 or by email at [Alisa.E.White.17@dartmouth.edu](mailto:Alisa.E.White.17@dartmouth.edu) or David A. Lutz, faculty advisor, by phone at (610) 350-9154 or by email at [David.A.Lutz@dartmouth.edu](mailto:David.A.Lutz@dartmouth.edu). This survey has been reviewed and approved by the Dartmouth College Institutional Review Board. If you have any questions about your rights as a participant, you may contact them by phone at (603) 646-6482.

I hope you enjoy completing the survey and I look forward to your response. By taking a few minutes to respond, you will help us a great deal with this study. As a token of our appreciation, you will be entered into a drawing for one of two $50 Home Depot cards upon completion of the survey.


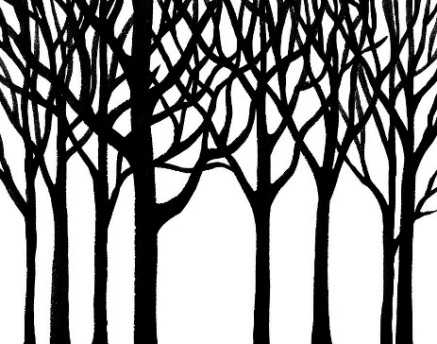


Many Thanks,

Alisa E. White

Dartmouth College Class of 2017

Environmental Studies Program

Senior Honors Thesis Candidate
