# Supplementary material for: Small-scale forestry and carbon offset markets: An empirical study of Vermont Current Use forest landowner willingness to accept carbon credit programs
Source: PLoS One. 2018 Aug 14;13(8):e0201967. doi: 10.1371/journal.pone.0201967 (PMC6091951; doi:10.1371/journal.pone.0201967)
Supplement: S3 Appendix — (DOCX) [file pone.0201967.s003.docx]

## **S3 Appendix. Mail Survey**

| 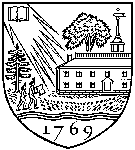 Dartmouth College  *Environmental Studies Program*  *6182 Steele Hall*  *Hanover, NH 03755-3571 USA*  **2017 Vermont**  **Current Use Forest**  **Landowner Survey**  **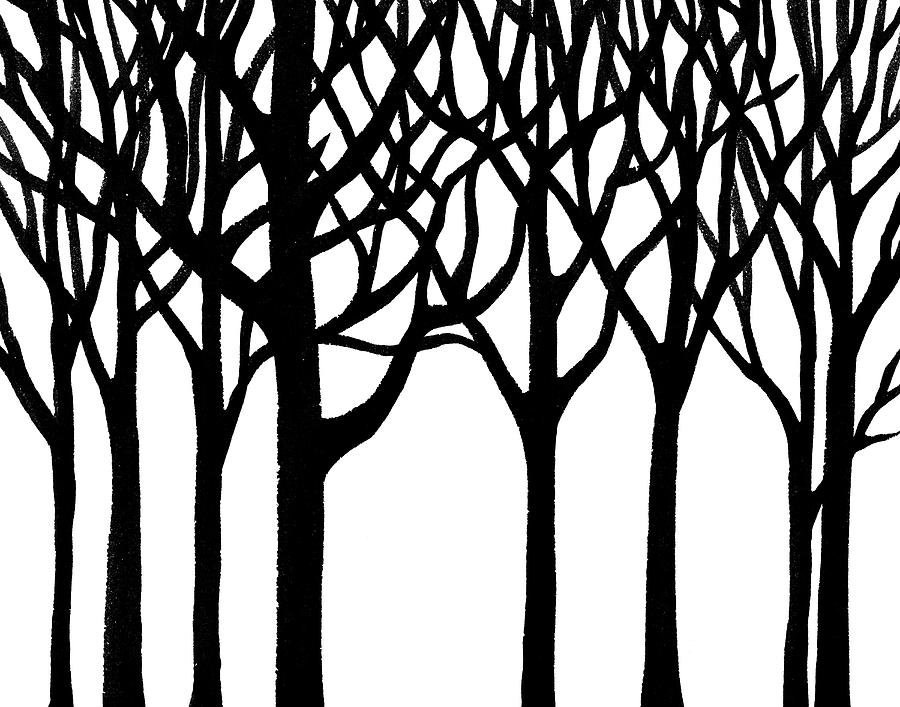**  **Survey Instructions:** This survey is part of a study about how forest landowners in Vermont’s Current Use Program would like to manage and generate income from their forests in the future. Please have an adult from your household, or, if applicable, a representative of your trust, estate, or business who is knowledgeable about your forest land in Vermont’s Current Use Program complete the survey.    Dartmouth College  *Environmental Studies Program*  *6182 Steele Hall*  *Hanover, NH 03755-3571 USA*   \|  \|  \|  \| \| --- \| --- \| --- \| |
| --- | --- | --- | --- |

| **Section 1 of 4: Questions About Your Forest Management and Ownership** |
| --- |

**Instructions:** Please answer the following questions about your forest management and ownership.

1. What category below best describes your ownership of your forest land in Vermont’s Current Use Program?

- Individual
- Joint, such as husband and wife
- Family partnership
- Trust or estate
- Corporation or business partnership
- Other (please specify):

___________________________________

1. How many acres of forest land do you have in Current Use?

|  |  |  |  |  |  | acres |
| --- | --- | --- | --- | --- | --- | --- |

1. How many distinct, unconnected properties of forest land do you have in Current Use?

|  |  |  |  |  |  | property/properties |
| --- | --- | --- | --- | --- | --- | --- |

1. In what year did you purchase/inherit your forest land held in Current Use?

|  |  |  |  |
| --- | --- | --- | --- |

1. Have you ever withdrawn your forest land from the Current Use program?

- Yes
- No

1. How close do you live to your forest land in Current Use? (check one)

- Less than 10 miles away
- 10-100 miles away
- Over 100 miles away

1. Do you have a forest management plan for your forest land in Current Use?

- Yes
- No

1. Do you plan on selling your Current Use forest land? (check one)

- In the next ten years
- In the next twenty years
- In the next fifty years
- I do not plan on selling my land
- I don’t know

1. How likely are you to put your forest land in Current Use under an easement in the next ten years? (check one)

- Extremely likely
- Somewhat likely
- Neither likely nor unlikely
- Somewhat unlikely
- Extremely unlikely

1. How important are the following as reasons for you to own your forest land in Vermont? *Mark only one box for each row.*

**Not Important……...Very Important**

|  | 1 | 2 | 3 | 4 | 5 |
| --- | --- | --- | --- | --- | --- |
| a) To enjoy beauty or scenery |  |  |  |  |  |
| b) To protect nature or biological diversity |  |  |  |  |  |
| c) As part of my home site/primary residence |  |  |  |  |  |
| d) As part of a cabin or vacation home site |  |  |  |  |  |
| e) To pass land on to children or other heirs |  |  |  |  |  |
| f) For firewood |  |  |  |  |  |
| g) For timber products (like logs or pulpwood) |  |  |  |  |  |
| h) For non-timber forest products (such as berries or maple syrup) |  |  |  |  |  |
| j) For hunting, fishing or other recreation |  |  |  |  |  |
| k) For privacy |  |  |  |  |  |

| 1. Please answer the following questions about your forest management activities on you forest land in Current Use.  \| **Yes** \| **No** \|  \| \| --- \| --- \| --- \| \|  \|  \| 1. Have any trees been harvested from your forest since you have owned it? \| \|  \|  \| 1. Have you worked with a consulting forester at all in the past five years? \| \|  \|  \| 1. Have you sold timber from your forest in the past five years? \| \|  \|  \| 1. Do you plan on harvesting any trees from your forest in the future? \| \|  \|  \| 1. Is any of your forest land under a conservation easement? \| \|  \|  \| 1. Have you ever worked with nearby landowners on forest management? \| \|  \|  \| 1. Are you a member of Vermont Woodlands Association? \| \|  \|  \| 1. Are you a member of a conservation organization? \| \|  \|  \| 1. Are you a member of Vermont Maple Sugar Makers Association? \| |
| --- | --- | --- | --- | --- | --- | --- | --- | --- | --- | --- | --- | --- | --- | --- | --- | --- | --- | --- | --- | --- | --- | --- | --- | --- | --- | --- | --- | --- | --- | --- |

| **Section 2 of 4: Questions About Your Views on Climate Change** |
| --- |

**Instructions**: Please respond to the following questions about your views on climate change. Climate change refers to the change in weather patterns resulting from increased carbon dioxide and other greenhouse gas emissions into the atmosphere.

1. Do you think that climate change is happening?

- Yes
- No

1. How confident are you about your response to the previous question (Q12)? (check one)

- I am extremely sure of this
- I am very sure of this
- I’m somewhat sure of this
- I’m not at all sure of this

1. Assuming climate change is happening, do you think

it is ... (check all that apply)

- - Caused mostly by human activities
  - Caused mostly by natural changes in the environment
  - Other
  - None of the above because climate change isn't happening

1. How worried are you about climate change?

(check one)

- - Very worried
  - Somewhat worried
  - Not very worried
  - Not at all worried

| \| **Section 3 of 4: Questions About Hypothetical Carbon Credit Programs** \| \| --- \|   **Instructions:** We are now going to look at some *hypothetical carbon credit programs*. Carbon credit programs, also known as carbon offset programs, can provide you additional income if you manage the trees in your forest to take in more carbon in the long run. Please read the frequently asked questions (FAQ) about these programs before evaluating the features of these programs in the following pages.  **Carbon Credit Programs FAQ**  What is forest carbon?   - Trees take in carbon from the air and store it in their trunks, leaves, and roots. - When trees absorb more carbon, there is less carbon dioxide in the atmosphere to contribute to climate change.   What are forest carbon credits?   - If you manage your forest to take in more carbon, you can apply to receive carbon credits for the additional carbon the trees store. - Through a carbon credit program, you can sell these carbon credits to a power plant, company, or other entity that produces too much carbon dioxide and wants to avoid receiving a fine for their emissions. |
| --- | --- |

**Carbon Credit Programs FAQ Continued**

What would I have to do to be part of a carbon credit program?

- You can keep your land in the Current Use program
- A forester will verify each year that you are complying with the program.
- **You are still allowed to harvest trees from your forest** but you must either: grow more trees per acre OR allow trees to grow until they are old.

What are the benefits of carbon credit programs?

- Carbon credit programs could give you additional annual income from your forest land.
- These programs may help reduce carbon dioxide levels in the atmosphere.

**Instructions:** Now, consider each of the following hypothetical carbon credit programs. For each program:

1. Consider all the features of the program and please mark **ONLY ONE** worst feature and **ONLY ONE** best feature of the program. The worst feature should be the program feature you like the least while the best feature should be the one you like the most of all.
2. Consider each carbon credit program **ON ITS OWN** when you decide if you would enroll in it if it were available to you. You can check “yes” for more than one program.

| Sample Carbon Credit Program Y is an example of how to fill in your responses for each program. Please fill in your own responses for every other program.  **Sample Carbon Credit Program Y**   \| **Worst feature**  **(Check one)** \| **Program Features** \| **Best feature**  **(Check one)** \| \| --- \| --- \| --- \| \|  \| Feature #1 \|  \| \|  \| Feature #2 \|  \| \|  \| Feature #3 \|  \| \|  \| Feature #4 \|  \| \| **Would you enroll in this program**  **if it were available to you? (check one)** \| \| \| \| - Yes   - - - No \| \| \|   **Carbon Credit Program 1**   \| **Worst feature**  **(Check one)** \| **Program Features** \| **Best feature**  **(Check one)** \| \| --- \| --- \| --- \| \|  \| Work with a not-for-profit organization to participate \|  \| \|  \| You will earn $15 per acre every year after costs \|  \| \|  \| You must stay in the program for 20 years \|  \| \|  \| You must pay a $100 per acre penalty if you withdraw early \|  \| \| **Would you enroll in this program**  **if it were available to you? (check one)** \| \| \| \| - - - - Yes       - No \| \| \| |  |
| --- | --- | --- | --- | --- | --- | --- | --- | --- | --- | --- | --- | --- | --- | --- | --- | --- | --- | --- | --- | --- | --- | --- | --- | --- | --- | --- | --- | --- | --- | --- | --- | --- | --- | --- | --- | --- | --- | --- | --- | --- | --- | --- | --- |
| **Carbon Credit Program 2**   \| **Worst feature**  **(Check one)** \| **Program Features** \| **Best feature**  **(Check one)** \| \| --- \| --- \| --- \| \|  \| Work with a for-profit company to participate \|  \| \|  \| You will earn $10 per acre every year after costs \|  \| \|  \| You must stay in the program for 60 years \|  \| \|  \| You must pay a $100 per acre penalty if you withdraw early \|  \| \| **Would you enroll in this program**  **if it were available to you? (check one)** \| \| \| \| - - - - Yes       - No \| \| \|   **Carbon Credit Program 3**   \| **Worst feature**  **(Check one)** \| **Program Features** \| **Best feature**  **(Check one)** \| \| --- \| --- \| --- \| \|  \| Work with a not-for-profit organization to participate \|  \| \|  \| You will earn $10 per acre every year after costs \|  \| \|  \| You must stay in the program for 100 years \|  \| \|  \| No penalty if you withdraw early \|  \| \| **Would you enroll in this program**  **if it were available to you? (check one)** \| \| \| \| - - - - Yes       - No \| \| \| |  |
| **Carbon Credit Program 4**   \| **Worst feature**  **(Check one)** \| **Program Features** \| **Best feature**  **(Check one)** \| \| --- \| --- \| --- \| \|  \| Work with a government organization to participate \|  \| \|  \| You will earn $5 per acre every year after costs \|  \| \|  \| You must stay in the program for 100 years \|  \| \|  \| You must pay a $100 per acre penalty if you withdraw early \|  \| \| **Would you enroll in this program**  **if it were available to you? (check one)** \| \| \| \| - - - - Yes       - No \| \| \|   **Carbon Credit Program 5**   \| **Worst feature**  **(Check one)** \| **Program Features** \| **Best feature**  **(Check one)** \| \| --- \| --- \| --- \| \|  \| Work with a for-profit company to participate \|  \| \|  \| You will earn $5 per acre every year after costs \|  \| \|  \| You must stay in the program for 20 years \|  \| \|  \| No penalty if you withdraw early \|  \| \| **Would you enroll in this program**  **if it were available to you? (check one)** \| \| \| \| - - - - Yes       - No \| \| \| |  |
| **Carbon Credit Program 6**   \| **Worst feature**  **(Check one)** \| **Program Features** \| **Best feature**  **(Check one)** \| \| --- \| --- \| --- \| \|  \| Work with a government organization to participate \|  \| \|  \| You will earn $15 per acre every year after costs \|  \| \|  \| You must stay in the program for 60 years \|  \| \|  \| No penalty if you withdraw early \|  \| \| **Would you enroll in this program**  **if it were available to you? (check one)** \| \| \| \| - - - - Yes       - No \| \| \|   **Carbon Credit Program 7**   \| **Worst feature**  **(Check one)** \| **Program Features** \| **Best feature**  **(Check one)** \| \| --- \| --- \| --- \| \|  \| Work with a government organization to participate \|  \| \|  \| You will earn $10 per acre every year after costs \|  \| \|  \| You must stay in the program for 20 years \|  \| \|  \| You must pay a $50 per acre penalty if you withdraw early \|  \| \| **Would you enroll in this program**  **if it were available to you? (check one)** \| \| \| \| - - - - Yes       - No \| \| \| |  |
| **Carbon Credit Program 8**   \| **Worst feature**  **(Check one)** \| **Program Features** \| **Best feature**  **(Check one)** \| \| --- \| --- \| --- \| \|  \| Work with a not-for-profit organization to participate \|  \| \|  \| You will earn $5 per acre every year after costs \|  \| \|  \| You must stay in the program for 60 years \|  \| \|  \| You must pay a $50 per acre penalty if you withdraw early \|  \| \| **Would you enroll in this program**  **if it were available to you? (check one)** \| \| \| \| - - - - Yes       - No \| \| \|   **Carbon Credit Program 9**   \| **Worst feature**  **(Check one)** \| **Program Features** \| **Best feature**  **(Check one)** \| \| --- \| --- \| --- \| \|  \| Work with a for-profit company to participate \|  \| \|  \| You will earn $15 per acre every year after costs \|  \| \|  \| You must stay in the program for 100 years \|  \| \|  \| You must pay a $50 per acre penalty if you withdraw early \|  \| \| **Would you enroll in this program**  **if it were available to you? (check one)** \| \| \| \| - - - - Yes       - No \| \| \| |  |

| **Section 4 of 4: Questions About You** |
| --- |

**Instructions:** Please answer the following questions about yourself.

1. How old are you? (check one)
   - Under 25 years
   - 25 to 34 years
   - 35 to 44 years
   - 45 to 54 years
   - 55 to 64 years
   - 65 to 74 years
   - 75 years and over
2. What is your gender?
   - Male
   - Female
3. What is the highest level of school you have completed? Please check only one. If you are currently in school, mark the highest degree received or grade completed.
   - Less than 12^th^ grade
   - High school graduate or GED
   - Some college
   - Associate or technical degree
   - Bachelor’s degree
   - Graduate degree
4. What is your household’s annual income? (check one)
   - Less than $25,000
   - $25,000 to $49,999
   - $50,000 to $99,999
   - $100,000 to $150,000
   - $150,000 to $200,000
   - $200,000 or more
   - Prefer not to answer
5. Are you of Hispanic or Latino origin?
   - Yes
   - No
6. What is your race? (check one or more)
   - American Indian or Alaska Native
   - Asian
   - Black or African American
   - Native Hawaiian or Other Pacific Islands
   - White
   - Prefer not to answer

# Would you be willing to participate in a follow up interview about your experience with this survey?

- - Yes
  - No

If yes, please put down the best way to contact

you for a follow up phone call:

_____________________________________

_____________________________________

_____________________________________

1. If you have any additional comments or concerns you would like to share about the survey, please share them in the blank space below.

| Thank you for participating in this survey. Please return your completed survey using the envelope with postage provided. If you would like a copy of the results of this survey, please print your name and an email address in the blank space below. If you do not have an email address, please write down your name and indicate you would like to receive the results by mail.  **Comments or questions about this survey?**  **Please Contact Us:**  Alisa E. White  Dartmouth College Environmental Studies Program  6182 Steele Hall  Hanover, NH 03755-3571 USA  Telephone: (518) 222-3743  Email: Alisa.E.White.17@dartmouth.edu  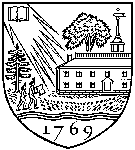 Dartmouth College  *Environmental Studies Program*  *6182 Steele Hall*  *Hanover, NH 03755-3571 USA* |
| --- |
