# Supplementary material for: Small-scale forestry and carbon offset markets: An empirical study of Vermont Current Use forest landowner willingness to accept carbon credit programs
Source: PLoS One. 2018 Aug 14;13(8):e0201967. doi: 10.1371/journal.pone.0201967 (PMC6091951; doi:10.1371/journal.pone.0201967)
Supplement: S4 Appendix — Following Dillman Tailored Mail Survey guidelines, we sent a follow up post card to remind landowners to complete the survey after it had been mailed to them. We also sent every landowner a follow up mail survey, identical to that shown in S3 Appendix, two weeks after this post card. (DOCX) [file pone.0201967.s004.docx]

## S4 Appendix. Follow-Up Post Card


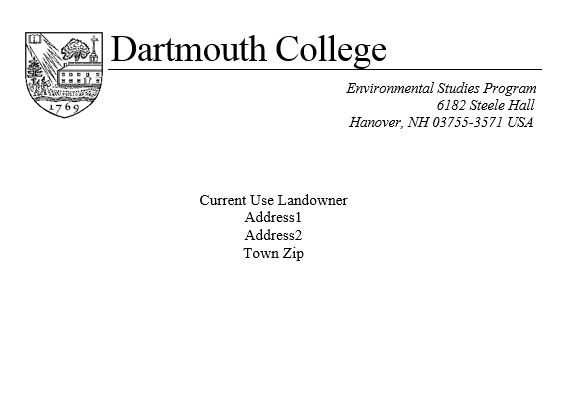


January 26, 2017

Last week, an important survey was mailed to your household or business because you were randomly selected to participate in a study about forest landowners in Vermont’s Current Use program.

If someone at this address has already completed and returned the survey, please accept our most sincere thanks. If not, please have an adult from your household or, if applicable, a representative or trustee do so as soon as possible. By completing this survey, you may help improve opportunities for forest owners in Vermont’s Current Use program. We are very grateful for your help with this study.

If you never received the survey or it was misplaced, please call us at 518-222-3743 or email us at [Alisa.E.White.17@dartmouth.edu](mailto:Alisa.E.White.17@dartmouth.edu) and we will mail you one promptly.

Sincerely,

Alisa E. White

Dartmouth College Class of 2017

Environmental Studies Program
